# Basal ganglia theta power indexes trait anxiety in people with Parkinson’s disease

**DOI:** 10.1093/brain/awae313

**Published:** 2025-04-03

**Authors:** Bart E. K. S. Swinnen, Colin W. Hoy, Elena Pegolo, Bryony Ishihara, Elena Ubeda Matzilevich, Julia Sun, Francesca Morgante, Erlick Pereira, Fahd Baig, Michael Hart, Huiling Tan, Zimi Sawacha, Martijn Beudel, Sarah Wang, Philip Starr, Simon Little, Lucia Ricciardi

**Affiliations:** 1 Department of Neurology, University of California San Francisco, San Francisco, CA 94158, USA; 2 Department of Neurology, Amsterdam University Medical Centers, Amsterdam Neuroscience, University of Amsterdam, Amsterdam 1105 AZ, The Netherlands; 3 Neurosciences and Cell Biology Institute, Neuromodulation and Motor Control Section, St George’s University of London, London SW17 0RE, UK; 4 Department of Information Engineering, University of Padova, Padova 35131, Italy; 5 Medical Research Council Brain Network Dynamics Unit, Nuffield Department of Clinical Neurosciences, University of Oxford, Oxford OX1 3TH, UK

**Keywords:** Parkinson’s disease, non-motor symptoms, deep brain stimulation, local field potential, anxiety

## Abstract

Neuropsychiatric symptoms are common and disabling in Parkinson’s disease, with troublesome anxiety occurring in one-third of patients. Management of anxiety in Parkinson’s disease is challenging, hampered by insufficient in-sight into underlying mechanisms, lack of objective anxiety measurements and largely ineffective treatments. In this study, we assessed the intracranial neurophysiological correlates of anxiety in patients with Parkinson’s disease treated with deep brain stimulation (DBS) in the laboratory and at home. We hypothesized that low-frequency (theta–alpha) activity would be associated with anxiety.

We recorded local field potentials from subthalamic nucleus or globus pallidus pars interna DBS implants in three Parkinson’s disease cohorts: (i) patients with recordings (subthalamic nucleus) performed in hospital at rest via peri-operatively externalized leads, without active stimulation, both ON and OFF dopaminergic medication; (ii) patients with recordings (subthalamic nucleus or globus pallidus pars interna) performed at home while resting, via a chronically implanted commercially available sensing-enabled neurostimulator (Medtronic Percept™ device), ON dopaminergic medication, with stimulation both on and off; and (iii) patients with recordings performed at home while engaging in a behavioural task via subthalamic nucleus and globus pallidus pars interna leads and electrocorticography paddles over the premotor cortex connected to an investigational sensing-enabled neurostimulator, ON dopaminergic medication, with stimulation both on and off.

Trait anxiety was measured with validated clinical scales in all participants, and state anxiety was measured with momentary assessment scales at multiple time points in the two at-home cohorts. Power in theta (4–8 Hz) and alpha (8–12 Hz) ranges was extracted from the local field potential recordings, and its relationship with anxiety ratings was assessed using linear mixed-effects models.

In total, 33 patients with Parkinson’s disease (59 hemispheres) were included. Across three independent cohorts, with stimulation off, basal ganglia theta power was positively related to trait anxiety (all *P* < 0.05). Also in a naturalistic setting, with individuals at home, at rest, with stimulation and medication ON, basal ganglia theta power was positively related to trait anxiety (*P* < 0.05). This relationship held regardless of the hemisphere and DBS target. There was no correlation between trait anxiety and premotor cortical theta–alpha power. There was no within-patient association between basal ganglia theta–alpha power and state anxiety.

We showed that basal ganglia theta activity indexes trait anxiety in Parkinson’s disease. Our data suggest that theta could be a possible physiomarker of neuropsychiatric symptoms and specifically of anxiety in Parkinson’s disease, potentially suitable for guiding advanced DBS treatment tailored to the needs of the individual patient, including non-motor symptoms.

## Introduction

People with Parkinson’s disease (PD) experience a range of non-motor symptoms in addition to their cardinal motor symptoms, with non-motor symptoms having a significant impact on the quality of life of both patients and caregivers.^1,2^ Among non-motor symptoms, neuropsychiatric deficits are prominent and can be present at any stage of the disease, even preceding motor deficits.^3^ Neuropsychiatric symptoms include depression, anxiety, apathy, psychosis, and impulse control disorders and related behaviours (ICB).^4^ The prevalence of neuropsychiatric symptoms in PD is high, reaching up to 50% for depression and anxiety.^5^

Anxiety has been recognized as one of the top three unmet therapeutic needs in PD patients and is recognized as a high research priority.^6^ Despite being so frequent, there are significant challenges in identifying, measuring and treating anxiety in PD.^7–10^ The mechanisms underlying anxiety in PD are unclear and often multifactorial, encompassing multiple disease-specific and individual-specific factors, which complicates the development of effective treatments. Anxiety in PD can be persistent, episodic or a combination of both, and anxiety can fluctuate during the day, influenced by intake of dopaminergic medication.^11^ The temporal dimensions of anxiety are often captured using two constructs: ‘trait’ anxiety, which constitutesgeneral level of anxiety of an individual over a considerable period, as opposed to ‘state’ anxiety, which reflects the momentary and dynamic anxiety level of a person.^12,13^ This aetiological and temporal variability complicates assessment of the presence and severity of anxiety, which currently relies on clinical interviews, clinician-administered scales and self-reported questionnaires. This lack of objective measures of anxiety makes the management of this common symptom highly challenging.^14,15^ Moreover, treatment options for anxiety in PD are currently limited, lack evidence-based support^16^ and are often ineffective.^17^ Altogether, anxiety management would greatly benefit from objective markers capable of indexing the presence and severity of anxiety, in addition to principled and targeted treatments.^7^

Deep brain stimulation (DBS) targeting either the subthalamic nucleus (STN) or the globus pallidus pars interna (GPi) is an effective treatment for motor symptoms in PD^18^ and might also improve non-motor symptoms,^19^ but the effect of DBS on neuropsychiatric symptoms and their fluctuations is still debated.^20^ Meta-analytical evidence suggests that, at the group level, anxiety symptoms improve after DBS.^21^ Electrophysiological activity, such as local field potentials (LFPs), can be recorded from the STN or GPi during DBS surgery, immediately postoperatively through externalized electrodes, and via chronically implanted sensing-enabled neurostimulators.^22^ The last method even allows for exploring physiological markers in the naturalistic environment, which is the most relevant setting for PD patients. By providing direct access to subcortical activity, LFP research has offered valuable insights into disease mechanisms and cognitive functions, including the identification of physiomarkers of PD motor symptom states that enable precise adjustments of therapy via adaptive DBS.^23–25^ These increasingly available techniques provide a unique opportunity to identify objective physiomarkers of neuropsychiatric symptoms, such as anxiety, that will be crucial for understanding and treating non-motor symptoms in PD.

Although subcortical beta (13–30 Hz) power has been established as a physiomarker for PD motor symptoms,^26^ the neural correlates of neuropsychiatric manifestations of PD are less well defined. Strikingly, although anxiety is a common and disabling symptom, the neural correlates of anxiety using basal ganglia electrophysiology have never been assessed. For other neuropsychiatric symptoms, studies assessing the associations between these symptoms and subcortical neural signals have been performed only in the hospital setting, by recording intra-operatively or immediately after surgery from externalized DBS electrodes during periods that can be confounded by microlesion effects.^27,28^ Increased power in the theta–alpha (4–12 Hz) band has been suggested as a physiomarker of neuropsychiatric symptoms in PD.^29^ Specifically, ICB^30^ and depression^31^ have been associated with increased STN theta–alpha and alpha power, respectively, and the severity of trait impulsivity has been positively related to STN alpha power.^32^ Other studies using behavioural paradigms have also implicated STN theta–alpha activity in emotional and behavioural processes.^29^

In the present study, we aimed to investigate the relationship between intracranial theta–alpha activity and anxiety in three separate cohorts of PD patients with STN and GPi DBS, both in hospital and at home, using investigational and commercially available sensing-enabled neurostimulators.

## Materials and methods

**Figure 1:**
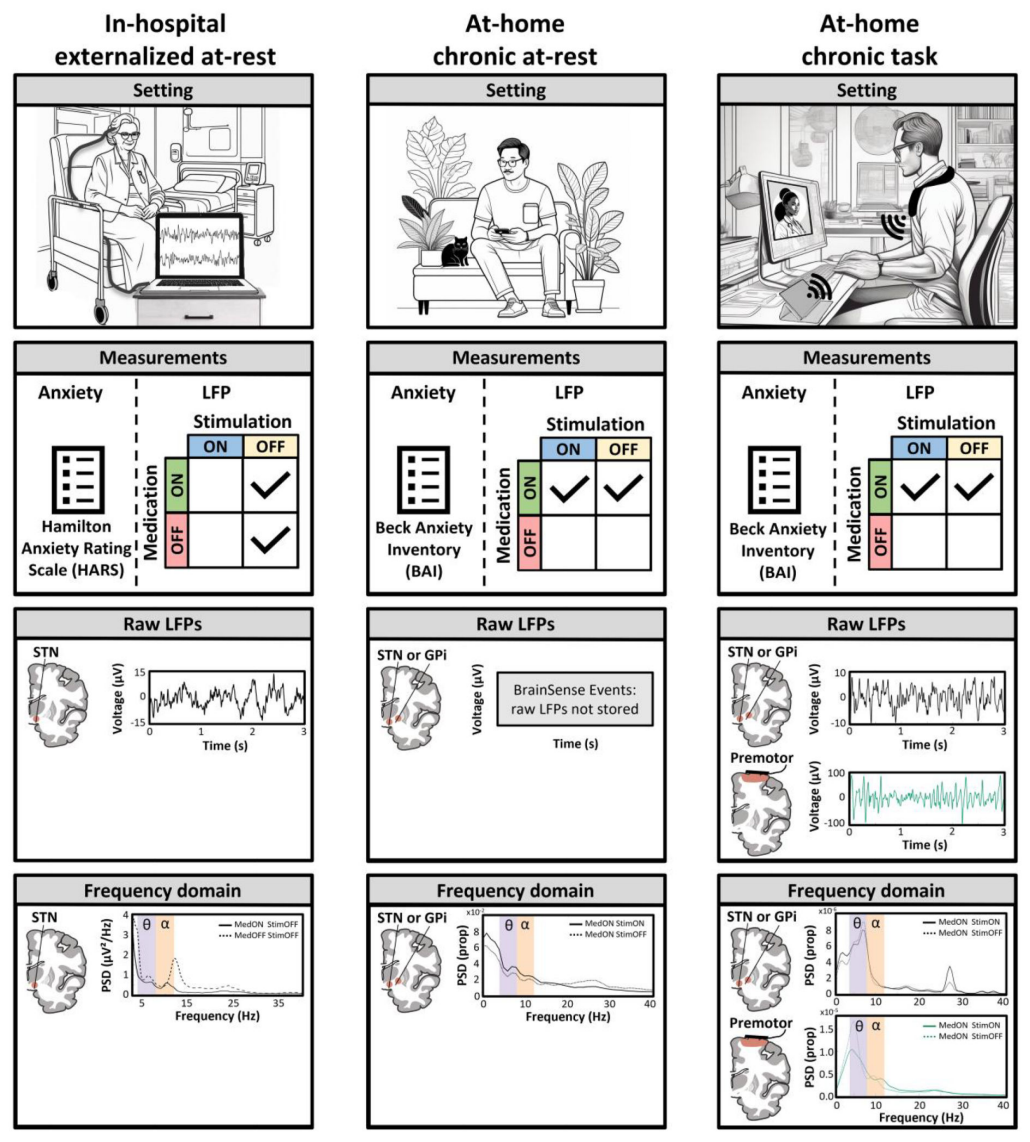
Three independent investigational cohorts for neurophysiological analysis. This study included three cohorts of Parkinson’s disease patients treated with deep brain stimulation. In the ‘in-hospital externalized at-rest’ cohort, basal ganglia LFP recordings were obtained from the STN a few days after surgery via externalized electrodes with the patient at rest. Patients were either OFF or ON medication, but stimulation was not yet enabled. Anxiety was rated with the Hamilton Anxiety Rating Scale (HARS). In the ‘at-home chronic at-rest’ cohort, basal ganglia (STN or GPi) LFPs were obtained at home in an unsupervised but semi-structured setting using the Percept PC’s BrainSense technology (BrainSense ‘Event’ triggered by the patient) with the patient at rest. Stimulation was either off or on, but patients were always in a medication ON state. Anxiety was rated with the Beck Anxiety Inventory (BAI). Raw LFPs were not available in the ‘at-home chronic at-rest’ cohort, whereas in the other two cohorts offline LFP processing was required to obtain frequency-domain data. In the ‘at-home chronic task’ cohort, basal ganglia (STN or GPi) and cortical (premotor cortex) LFPs were obtained via remotely supervised streaming via the Summit RC+S system with the patient at rest between trials of a behavioural task. Experimental conditions involved medication ON–stimulation on and medication ON–stimulation off. Anxiety was rated with the BAI. In all three cohorts, analyses were focused on the relationship between anxiety measures and power in the theta and alpha frequency bands. Infographics in the first row have been generated with the help of the generative artificial intelligence platform Stable Diffusion Online (https://stablediffusionweb.com/). LFP = local field potential; GPi = globus pallidus pars interna; PSD = power spectral density; STN = subthalamic nucleus.

Three independent PD cohorts have been included and assessed in the present study (Fig. 1): (i) the ‘in-hospital externalized at-rest’ cohort, with in-hospital LFP measurements via externalized DBS electrodes in patients with bilateral STN-DBS (London, UK); (ii) the ‘at-home chronic at-rest’ cohort, with at-home unsupervised LFP measurements through the chronically implanted Medtronic© Percept™ PC neurostimulator in patients with STN-DBS and GPi-DBS (San Francisco, CA, USA); and (iii) the ‘at-home chronic task’ cohort, with at-home supervised LFP measurements through the chronically implanted Medtronic© Summit RC+S neurostimulator in patients with STN-DBS and GPi-DBS performing a reward learning task (San Francisco). Data collection was performed independently for each of the three cohorts. Data were then collated and analysed simultaneously for all three cohorts to test for both statistical reproducibility and generalizability.

### Patients

#### In-hospital externalized at-rest cohort

Consecutive PD patients (*n* = 12) undergoing bilateral STN-DBS were recruited at St. George’s Universtiy Hospital, London, UK. Inclusion criteria were standard clinical criteria for DBS.^33^ In detail, they were as follows: (i) diagnosis of idiopathic PD; (ii) age <70 years; (iii) bothersome motor fluctuations and/or levodopa-induced dyskinesias despite optimal pharmacological management; (iv) absence of dementia, major depression with suicidal thoughts or acute psychosis; (v) significant clinical response to levodopa challenge (≥30% improvement in Movement Disorders Society-Unified Parkinson’s Disease Rating Scale III score); and (vi) disease duration >5 years.

#### At-home chronic at-rest cohort

Patients (*n* = 13) were recruited at the University of California San Francisco (UCSF), San Francisco, CA, USA. Inclusion criteria were as follows: (i) patients with idiopathic PD treated with unilateral or bilateral DBS of the STN or the GPi; (ii) implanted with the Percept™ PC neurostimulator; (iii) on relatively stable DBS parameters and PD medication (typically >6 months after surgery); and (iv) stimulation parameters in at least one hemisphere compatible with BrainSense™.

#### At-home chronic task cohort

Patients (*n* = 8) were recruited at UCSF. Note that this cohort involves a different sample of patients and different neurostimulator devices from the patients in the ‘at-home chronic at-rest’ cohort. Patients were part of a clinical trial involving PD patients undergoing DBS implantation for motor fluctuations (NCT03582891). These patients were implanted bilaterally (except for one patient with unilateral DBS) with subcortical leads (Medtronic© models 3389 and 3387) in either the STN or the GPi, in addition to quadripolar electrocorticography paddles (Medtronic© model 0913025) over the sensorimotor cortex. For each hemisphere, these electrodes were connected to an investigational sensing-enabled chronically implanted neurostimulator (Medtronic© Summit RC+S model B35300R^34^). Patients with bilateral DBS were therefore implanted with two neurostimulators.

### Data collection

#### In-hospital externalized at-rest cohort

Data were acquired 3–5 days after surgical insertion of the DBS leads, before the implantation of the neurostimulator, while patients were admitted to hospital. Recording of STN LFPs (*n* = 24 hemispheres) was performed with a TMSi-Porti amplifier (TMS International; frequency sampled at 2048 Hz), while participants were resting with eyes open, comfortably sitting on a chair for 5 min. The LFPs were recorded from the four contacts on the left and right of the bilateral electrodes. The bipolar signals (i.e. L0–L1, L1– L2, L2–L3, R0–R1, R1–R2 and R2–R3) were used for LFP analysis. Recordings were performed in two sessions on the same day: (i) in the morning, after overnight withdrawal of antiparkinsonian medication (Medication OFF condition); and (ii) in the clinically defined ON medication condition, 1 h after the administration of the regular dose of levodopa to a participant (MedON). The presence and severity of anxiety and depression were assessed using the Hamilton Anxiety Rating Scale (HARS)^35^ and Hamilton Depression Rating Scale (HDRS),^36^ respectively, on the day of the recording. The severity of motor symptoms and dyskinesia was assessed by means of the Unified Parkinson’s Disease Rating Scale (UPDRS) part III and the Unified Dyskinesia Rating Scale (UDRS) part III ‘Objective Impairment’ on the day of the study.

#### At-home chronic at-rest cohort

All participants were assessed in hospital at baseline. During this visit, demographic and clinical data were gathered, and the presence and severity of anxiety and depression were rated using the Beck Anxiety Inventory (BAI)^37^ and Beck Depression Inventory (BDI),^38^ respectively. BrainSense™ was enabled in the clinically active stimulation group on the Percept™ PC neurostimulator. To this end, sensing was activated in a sandwiched configuration around the active stimulation contact, located in the STN (*n* = 14 hemispheres) or GPi (*n* = 6 hemispheres). Using the BrainSense™ Event feature, an event called ‘Research’ was enabled for the patient. When an event is triggered by the patient using the patient programmer, a 30 s LFP recording is performed, the power–frequency spectrum of which is stored on the neurostimulator. Patients were instructed on the study protocol and how to use the patient programmer for the study at home. Patients performed research activities (see below) at home for a total of 14 days, in a structured but self-supervised manner. The timing of study activities was consistent within each patient and in the medication ON (MedON) state (i.e. 1 h after taking a dose of levodopa). Patients performed two consecutive rounds of study activities on a research day: once with active stimulation at clinical amplitude (MedON-StimOn) and once with stimulation at 0.0 mA (MedON-StimOff). The order of these two activities differed between days and was pseudorandomized across patients. One round of research activities involved the following consecutive activities: (i) setting the stimulation at clinical amplitudes or 0.0 mA using the patient programmer; (ii) resting for 1 min; (iii) triggering a ‘Research’ event using the patient programmer; (iv) resting for 1 min; (v) rating current anxiety state on a visual analogue scale (VAS), on which 0 = ‘not anxious at all’ and 100 = ‘very anxious’, and rating current mood state on a VAS (0 = ‘not depressed at all’ and 100 = ‘very depressed’); (vi) rating current severity of motor symptoms on a VAS (‘How are your Parkinson’s disease motor symptoms at the moment?’), on which 0 = ‘mild’ and 100 = ‘severe’; and (vii) completion of a behavioural paradigm (to be reported separately). After completing the at-home research activities, data were downloaded (i.e. JSON file export) from the neurostimulator in the hospital at their next visit.

#### At-home chronic task cohort

Data were collected during a previously conducted but unpublished study investigating neural correlates of value-based decision-making and momentary assessments of mood and anxiety. Participants performed a modified two-step reward learning task under remote supervision from the researcher while in their homes.^39^ Each participant performed this task between 9 and 13 times (*n* = 78 total sessions) across 5–7 days. VAS ratings of anxiety and happiness (‘how anxious are you at the moment?’, where 0 = ‘not at all anxious’ and 100 = ‘very anxious’; and ‘how happy are you at this moment?’, where 0 = ‘very unhappy’ and 100 = ‘very happy’). were collected immediately before starting the instruction phase of each task run. Experiments were performed in two clinical conditions: MedON-StimOn and MedON-StimOff, with at least two sessions per condition. Two patients could not tolerate the DBS therapy being completely off and were instead recorded at 53%/50% and 92%/92% of their clinical amplitude in the left/right hemispheres. The LFP data were recorded during each task session subcortically from the pair of contacts surrounding the clinical stimulation contact (i.e. sandwich configuration) in the STN (*n* = 8 hemispheres) or GPi (*n* = 7 hemispheres) and cortically from bipolar pairs of the two most anterior and two most posterior electrocorticography paddle contacts. All electrocorticography paddle contacts contributing to the cortical recording pairs reported here were located anterior to the central sulcus, ranging from the precentral gyrus to the middle/superior frontal gyrus. Posterior pairs were not analysed owing to between-subject variability in anatomical localization relative to the central sulcus. At baseline, the presence and severity of anxiety and depression were evaluated using the BAI and BDI.

### Data analysis

#### In-hospital externalized at-rest cohort

All the acquired LFPs originated from a quadripolar electrode. The bipolar arrangement from the acquired data was obtained offline. In this way, each electrode presented three bipolar signals. Data were first inspected visually, and parts of the signal with marked artefacts (e.g. signal saturation or movement artefacts) were removed {final signal length [mean ± standard deviation (SD)]: 274.0 ± 77.5 s}. Signals were then detrended and highpass filtered at 1 Hz (Butterworth). Spectral analysis was performed by computing the power spectrum of the signals using Welch’s method (2 s Hanning’s windows, 50% overlap) with a frequency resolution of 0.25 Hz. Theta and alpha bands were computed by averaging the band power between 4–8 and 8–12 Hz, respectively.

#### At-home chronic at-rest cohort

The LFP data from the ‘Research’ events for the 14 days of at-home assessments were retrieved from the JSON files. Within each ‘Research’ event, LFP data were proportionally normalized per frequency band by dividing the sum of powers per canonical frequency band (i.e. theta 4–8 Hz or alpha 8–12 Hz) over the sum of powers of the frequency range from 0 to 57.62 Hz. This upper limit was implemented because the powers above a varying frequency are censored by the processing onboard Percept PC, the cut-off of which depends on the stimulation frequencies (e.g. frequencies of >57.62 Hz are censored when stimulating at 180 Hz). For group-level comparisons of baseline BAI and BDI scores or average VAS anxiety with alpha or theta power, proportionally normalized powers per frequency band were averaged across events within hemispheres.

#### At-home chronic task cohort

The LFP data during task sessions were reconstructed using the *processRCS* analysis toolbox.^40^ Data were extracted from 0.9 s epochs when participants were resting during the intertrial interval. Trials were excluded for missing behavioural and/or neural data and practice trials (mean ± SD: 840.6 ± 165.0 trials per person). Power spectral density estimates were extracted from these time series using Welch’s method (*pwelch* in MATLAB), with 0.4 s windows and 50% overlap for frequencies ranging from 1 to 55 Hz in 0.5 Hz steps. To eliminate differences between subjects and regions, the power spectral densities were then normalized for each trial by dividing by the sum of all power bands from 1 to 55 Hz. Normalized power spectral densities were then averaged across trials within each session before computing theta and alpha power as the mean within 4–8 and 8–12 Hz bands, respectively. Theta and alpha power were then averaged across runs within each participant, yielding one estimate for each hemisphere within each participant.

#### Location of DBS electrodes

Details on reconstruction of the electrodes location and positioning of contacts are provided in the Supplementary material and Supplementary Fig. 1.

### Statistical analyses

Because data were structured with a high level of non-independence, especially concerning patients with bilateral LFP recordings, linear mixed-effects (LME) models were used to assess the relationship between the power of LFP frequency bands and behavioural measures (i.e. HDRS, HARS, BAI, BDI, VAS anxiety and VAS motor). The LME models predicted neural power averaged within each hemisphere of each patient using fixed effects of each participant’s symptom score, hemisphere (left/right), basal ganglia region (STN/GPi) and random intercepts for each participant. To control for the effect of motor symptoms, including tremor and dyskinesia, on theta and alpha power, in the ‘in-hospital externalized at-rest’ cohort, a secondary analysis was conducted with an additional LME model, adding the fixed effect of the presence of either tremor or dyskinesia (according to UPDRS III or UDRS part III scores) to the main model. Separate models were used to predict either theta or alpha power using one of the *a priori* defined symptom metrics. One-sided likelihood ratio tests were used to assess the significance of the relationships by using the *compare* function in MATLAB R2023a (Mathworks, Natick, MA, USA), testing whether the model including the fixed effect of the symptom predict- or of interest (e.g. BAI) improved model fit at a *P*-level defined at 0.05 in comparison to a null model without that predictor. The same LME model structure was used to assess state anxiety by using VAS anxiety ratings for individual events in the ‘at-home at-rest’ cohort and runs in the ‘at-home task’ cohort to predict low-frequency power, again with separate models for theta and alpha power.

### Ethical approval

All patients provided written informed consent under the Declaration of the Principles of Helsinki. For the London cohort, approval was obtained from the Institutional Review Board of the Integrated Research Application System (IRAS), with the study protocol number 268941 (‘Neural basis of neuropsychiatric symptoms in PD’). For the UCSF cohorts, approval was obtained from the Institutional Review Board of the UCSF (study numbers 10-01350 and 20-31239, respectively).

### Citation diversity statement

Given recent work identifying biases in citation practices such that papers from women and other minority scholars are under-cited relative to the number of such papers in the field,^41–44^ we sought pro-actively to consider choosing references that reflect the diversity of the field. To support equitable practices in science, we report the predicted gender and racial/ethnic category of the first and last author of each reference using databases that store the probability of a first name being carried by a woman^41^ or a person of colour,^45,46^ alongside the details of these methods and their limitations (Supplementary material).

## Results

Thirty-three participants (59 hemispheres) were included: 12 in the ‘in-hospital externalized at-rest’ cohort, 13 in the ‘at-home chronic at-rest’ cohort, and 8 in the ‘at-home chronic task’ cohort. Demographics and clinical data are displayed in Table 1 and in the Supplementary material, Results.

### Effect of medication and DBS on low-frequency oscillations

To evaluate the effect of medication (‘in-hospital externalized at-rest cohort’) or stimulation (in the presence of dopaminergic medication, the two ‘at-home’ chronic cohorts) on the power frequency spectrum, we evaluated an LME model predicting the power of the band under analysis (theta, alpha, low-beta, high-beta and gamma) using fixed effects of each participant’s hemisphere (left/right), basal ganglia region (STN/GPi; only in San Francisco cohorts with both regions) and random intercepts for each participant. A separate model adding the fixed effect of medication (London cohort) or stimulation (San Francisco cohorts) was computed and compared with the null model using one-sided likelihood ratio tests via the *compare* function in MATLAB R2023a (Mathworks) with the *P*-value set at 0.05.

In the ‘in-hospital externalized at-rest cohort’, we observed trends towards a statistically significant effect of the medication on theta (β = 0.654; *P* = 0.05) and low-beta (β = −1.004; *P* = 0.07) bands, whereas no statistically significant effect was found on alpha, high-beta and gamma bands (all *P* > 0.42) (Supplementary Fig. 2A).

In the ‘at-home chronic at-rest cohort’, results of this analysis show that stimulation has no effect on the power in the theta (*P* = 0.9574) and alpha (*P* = 0.7465) frequency ranges, but decreases the power in the low-beta, high-beta and gamma frequency ranges (β = −0.4887, −0.3689 and −0.0639; all *P* < 0.0001; Supplementary Fig. 2B).

In the ‘at-home chronic task cohort’, stimulation had no effect on basal ganglia power in any frequency band (all *P* > 0.147; Supplementary Fig. 2C).

### Effect of DBS on anxiety and mood

We assessed the acute effect of DBS on anxiety and mood in the ‘at-home chronic at-rest’ and ‘at-home chronic task’ cohorts, as measured with VAS anxiety and VAS mood (‘happiness’ in the ‘at-home chronic task’ cohort). We performed LMEs with fixed effects of DBS status and basal ganglia region of interest and random intercepts by subject to predict daily VAS ratings of symptoms. In the ‘at-home chronic at-rest cohort’, VAS scores of anxiety were lower with MedON-StimOn (mean ± SD: 23.5 ± 19.2) compared with MedON-StimOff (28.9 ± 23.1; β = 13.3, *P* < 0.0001). VAS scores of mood were also better with MedON-StimOn (18.4 ± 17.1) compared with MedON-StimOff (20.3 ± 19.1; β = 10.7, *P* < 0.0001).

In the ‘at-home chronic task cohort’, there was no effect of DBS on the VAS anxiety scores (β = −4.44, *P* = 0.311; mean ± SD: 38.9 ± 21.3 StimOn, 43.4 ± 24.3 StimOff). VAS happiness ratings were significantly higher with StimOn (68.2 ± 16.2) compared with StimOff (63.3 ± 21.5; β = 5.05, *P* = 0.048).

### Relationship between trait anxiety and neurophysiological measures

**Figure 2:**
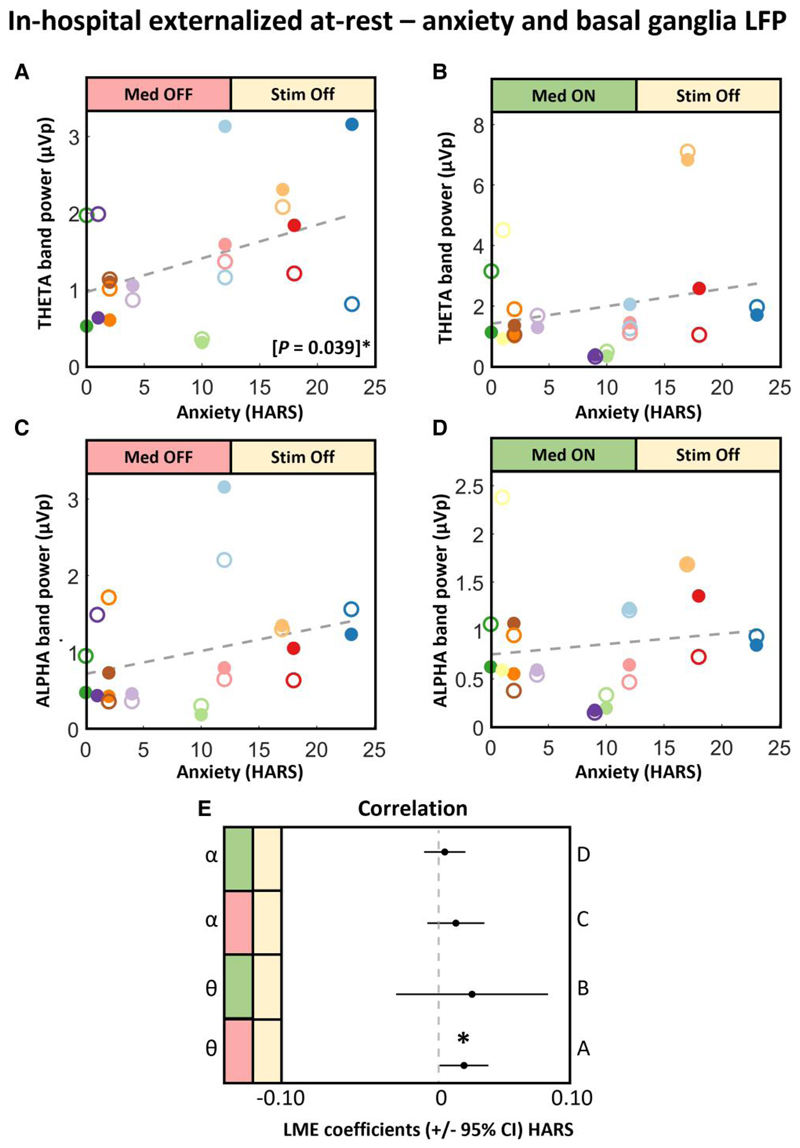
Anxiety is related to basal ganglia theta in in-hospital externalized recordings. This figure displays the relationship between anxiety measured by the HARS and basal ganglia (STN) theta and alpha band power in the ‘in-hospital externalized at-rest’ cohort. Each colour represents a patient (*n* = 12 patients), and empty/filled markers indicate right/left hemisphere recordings (*n* = 24 hemispheres). (**A**) With medication OFF–stimulation off, a positive relationship is present between anxiety and basal ganglia theta. (**B**–**D**) No relationship between anxiety and theta is present with medication ON-stimulation off; no relationship between anxiety and alpha in any condition. (**E**) Coefficients of anxiety in LME models. *P < 0.05. CI = confidence interval; HARS = Hamilton anxiety rating scale; LME = linear mixed-effects; STN = subthalamic nucleus.

Initially, we aimed to assess whether the low-frequency bands, which have been implicated in neuropsychiatric/cognitive aspects of PD, are also associated with anxiety, using LFP recordings obtained shortly after surgery via externalized leads. In the ‘in-hospital externalized at-rest’ cohort, anxiety (as per HARS score) was positively related to STN theta power with MedOFF-StimOff (*P* = 0.039, β = 0.044), and this relationship was trending towards significant with MedON-StimOff (*P* = 0.370, β = 0.058) (Fig. 2A, B and E). Anxiety was not related to STN alpha power in either condition (Fig. 2C–E). No significant effect of laterality was found (right/left hemisphere) with either theta or alpha power (all *P* > 0.05). The secondary analysis adding tremor (seven patients with tremor in MedOFF-StimOff, three patients with tremor in MedON-StimOff) or dyskinesia (eight patients with dyskinesia in MedON-StimOff) as fixed effects to the main models showed no association between tremor and alpha or theta and between dyskinesia and alpha or theta (all *P* > 0.05), and the relationship between theta and anxiety held when controlling for these motor symptoms in the model (*P* = 0.04, β = 0.041).

**Figure 3:**
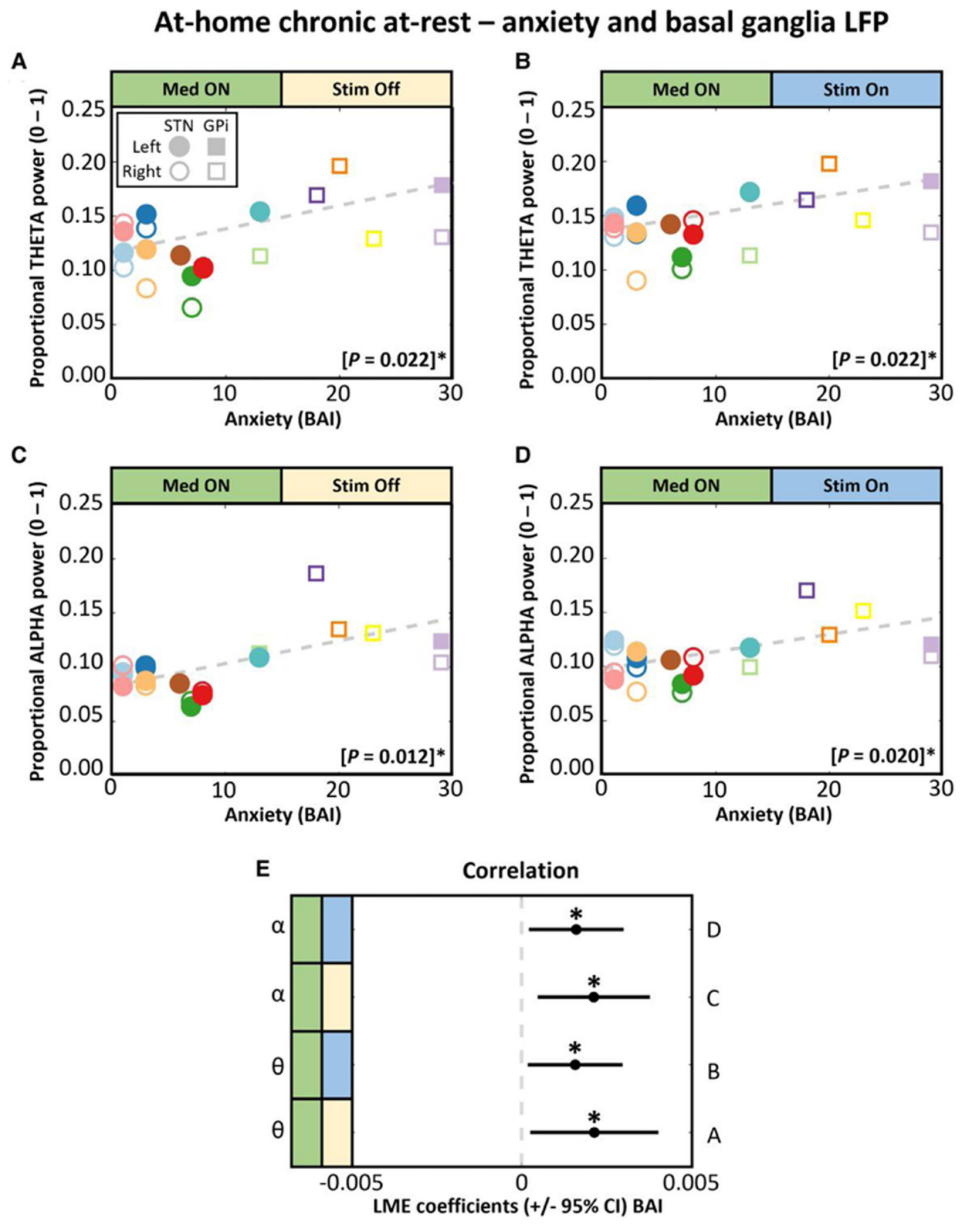
Anxiety is related to basal ganglia theta and alpha in at-home at-rest recordings. Relationship between anxiety and subcortical LFP in the ‘at-home chronic at-rest’ cohort. Scatter plot colours indicate the participants (*n* = 13 patients), with circles indicating STN implants and squares GPi implants. Empty/filled markers indicate right/left hemisphere recordings (*n* = 19 hemispheres). Theta and alpha band power were extracted from at-home at-rest BrainSense Event LFP recordings, and correlation was assessed with baseline anxiety measured with the BAI. With medication ON–stimulation off (**A** and **C**) and medication ON–stimulation on (**B** and **D**), anxiety is positively related to subcortical theta (**A** and **B**) and alpha (**C** and **D**) band power. (**E**) Coefficients of BAI in LME models. *P < 0.05. BAI = Beck Anxiety Inventory; CI = confidence interval; GPi = globus pallidus pars interna; LFP = local field potential; LME = linear mixed-effects model; STN = subthalamic nucleus.

**Figure 4:**
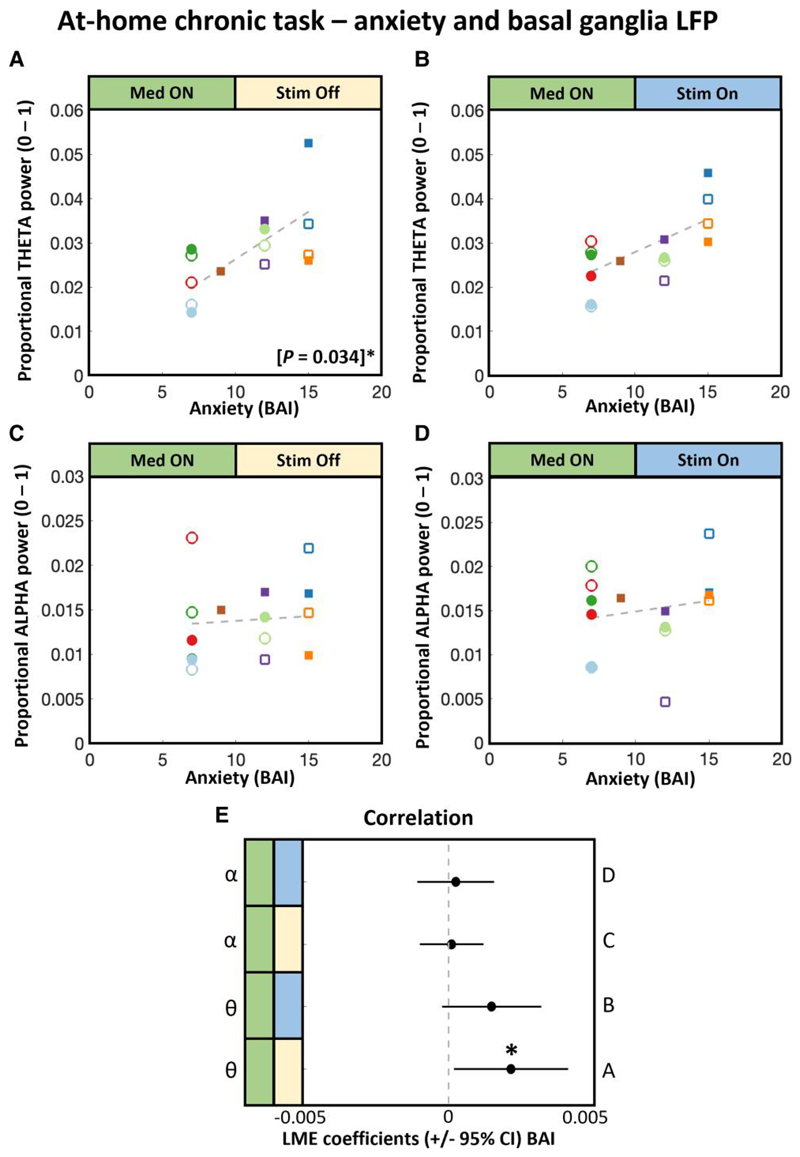
Anxiety is related to basal ganglia theta in at-home recordings during a cognitive task. Relationship between anxiety and subcortical LFP during a reward task in the ‘at-home chronic task’ cohort. Theta and alpha power were extracted from the intertrial interval in a reward task, and correlation was assessed with baseline anxiety (BAI). Scatter plot colours indicate participants (*n* = 8 patients), with circles indicating STN implants and squares GPi implants. Empty/filled markers indicate right/left hemisphere recordings (*n* = 15 hemispheres). With medication ON–stimulation off (**A**), there is a positive relationship between anxiety and subcortical theta band power. (**B**–**D**) No relationship between anxiety and theta is present with medication ON-stimulation on; no relationship between anxiety and alpha in any condition. (**E**) Coefficients of BAI in LME models. *P < 0.05. BAI = Beck Anxiety Inventory; CI = confidence interval; GPi = globus pallidus pars interna; LFP = local field potential; LME = linear mixed-effects model; STN = subthalamic nucleus.

Next, we investigated the relationship between anxiety and low-frequency bands when recording LFPs at home from patients with chronically implanted neurostimulators equipped with sensing capabilities. In the ‘at-home chronic at-rest’ cohort with unsupervised LFP recordings (mean = 21.9, SD = 5.4 BrainSense™ Event recordings per patients), anxiety (according to BAI score) was positively related to basal ganglia theta power in both the MedON-StimOff (*P* = 0.022, β = 0.0021) and MedON-StimOn (*P* = 0.022, β = 0.0016) conditions (Fig. 3A, B and E). Moreover, in this cohort, anxiety was also positively related to basal ganglia alpha power in both conditions (MedON-StimOff: *P* = 0.012, β = 0.0021; MedON-StimOn: *P* = 0.020, β = 0.0016; Fig. 3C–E). In the ‘at-home chronic task’ cohort, with supervised LFP recordings during a reward learning task, anxiety (according to BAI score) was positively related to basal ganglia theta power in the MedON-StimOff condition (*P* = 0.034, β = 0.0022), and this relationship was trending towards significant with MedON-StimOn (*P* = 0.079, β = 0.0015) (Fig. 4A, B and E).

We did not find any association between anxiety and basal ganglia alpha power in either at-home cohort (Fig. 4C–E).

Altogether, a positive relationship between anxiety and basal ganglia theta power was present consistently across three independent cohorts in both subacute clinical and naturalistic environments.

We also aimed to assess whether the relationship between anxiety and theta is specific to the basal ganglia structures targeted with DBS (i.e. STN and GPi), in comparison to anatomically connected premotor cortical areas. In the ‘at-home chronic task’ co-hort, electrocorticography data over the premotor cortex showed that anxiety was unrelated to cortical theta or alpha power (all *P* > 0.560; Supplementary Fig. 3). Of note, the involvement of other prefrontal–limbic–temporal cortical areas that have previously been implicated in a non-motor-related theta network could not be assessed in this cohort.^47^

### Assessing contributions of state anxiety and motor symptoms

Next, we aimed to investigate whether, beyond indexing ‘trait’ anxiety (e.g. HARS and BAI), basal ganglia theta power could also be related to within-subject variations in ‘state’ anxiety. This was assessed in the at-home cohorts. For the ‘at-home chronic rest’ cohort, for a period of ~14 days, patients provided daily reports of ecological momentary assessments of their anxiety level on a VAS immediately after triggering an LFP recording. At the within-subject level, VAS anxiety (representing ‘state’ anxiety) was not related to basal ganglia theta or alpha power, in either MedON-StimOff (*P* = 0.147 and β < 0.0001 for theta, *P* = 0.832 and β < 0.0001 for alpha) or MedON-StimOn conditions (*P* = 0.960 and β < 0.0001 for theta, *P* = 0.870 and β < 0.0001 for alpha) (Supplementary Fig. 4A–E). Likewise, VAS anxiety ratings did not predict low-frequency power in the ‘at-home chronic task’ cohort. Specifically, VAS anxiety ratings were acquired immediately before starting the task, to provide a measure of anxiety fluctuations across days, and as in the ‘at-home chronic at-rest cohort’, VAS anxiety scores at the run level did not predict either theta or alpha power in MedON-StimOff or MedON-StimOn conditions (all *P* > 0.143, all β < 0.0001) (Supplementary Fig. 4F–J). This suggests that within-subject ‘state’ anxiety fluctuations are not directly related to basal ganglia theta power.

In contrast, in the ‘at-home chronic rest’ cohort, averaging VAS anxiety scores per patient across 14 days to derive an estimate of ‘trait’ anxiety revealed a positive relationship to basal ganglia theta power in MedON-StimOff (*P* = 0.023, β = 0.0008) and was trending towards significant in MedON-StimOn (*P* = 0.051, β = 0.0007) (Supplementary Fig. 5A, B and E). Average VAS anxiety per patient was also positively related to basal ganglia alpha power, with MedON-StimOff (*P* = 0.012, β = 0.0009) and MedON-StimOn (*P* = 0.032, β = 0.0007) (Supplementary Fig. 5C–E). Thus, ‘trait’ measures of anxiety computed by averaging VAS anxiety ratings across days also predict theta and alpha power, which aligns with our core finding of a relationship between low-frequency power and ‘trait’ anxiety measured by surveys (i.e. HARS and BAI). In sum, basal ganglia theta and alpha power reflected the average VAS anxiety ratings across days in the ‘at-home chronic at-rest’ cohort, but did not track day-to-day fluctuations in anxiety in either at-home cohort, indicating a stronger correspondence to ‘trait’ than ‘state’ anxiety levels.

Given that anxiety fluctuations during the day are often associated with changes in the motor state of a patient, we then explored whether changes in anxiety were influenced by changes in the overall motor state as perceived by the patient using VAS motor symptoms ratings. We analysed concurrent measures of motor symptoms and anxiety from the ‘at-home chronic at-rest cohort’ using VAS ratings (as detailed in the Materials and Methods). We found a positive relationship within subject between daily VAS anxiety and VAS motor scores in both MedON-StimOff (β = 0.3341, *P* = 0.0018) and MedON-StimOn (β = 0.4866, *P* < 0.0001) states. These findings validate the previously reported relationship between anxiety and motor functions in PD.^48^

We next examined whether these motor symptoms predicted basal ganglia activity. Using VAS motor ratings averaged within each patient to predict neural power, we found that average VAS motor scores were inversely related to basal ganglia theta in the MedON-StimOff conditions (β = −0.0007, *P* = 0.0231) but not in the MedON-StimOn conditions (β = 0.0003, *P* = 0.6340). No significant relationship was found between average VAS motor scores and basal ganglia alpha activity.

We also tested whether the relationship between anxiety and basal ganglia theta power was affected by VAS motor symptoms scores by including motor and anxiety VAS scores as fixed effects in the same LME model. We found that average VAS anxiety continued to predict basal ganglia theta power (β = 0.0012, *P* = 0.0004) in the MedON-StimOff state and a trend toward statistical significance in the MedON-StimOn state (β = 0.0007, *P* = 0.0890) in this combined model.

In summary, although daily fluctuations in anxiety and motor symptoms are correlated within subjects, trait anxiety (average VAS anxiety) remains a robust predictor of subcortical theta power, even when controlling for overall motor symptom severity (average VAS motor).

Lastly, we explored the relationship between depression and low-frequency oscillations,^31^ but found inconsistent results across the three cohorts (Supplementary material and Supplementary Fig. 6).

In summary, our study revealed a consistent positive association between anxiety and basal ganglia theta power in PD patients across different cohorts and conditions, indicating a potential neural correlate of anxiety in PD. Importantly, even when accounting for over-all motor symptom severity, whether assessed in the clinic using clinical scales or at home with VAS ratings, trait anxiety remains a robust predictor of basal ganglia theta power. This underscores that the relationship between trait anxiety and subcortical theta activity is independent of the severity of motor symptoms.

## Discussion

In the present study, we evaluated the basal ganglia neural correlates of anxiety in PD patients for the first time, using both in-laboratory and at-home recordings. Across three independent cohorts, our results demonstrate that subcortical theta power recorded from STN and GPi is positively related to anxiety in PD, with higher theta power indexing more severe anxiety. This effect was present regardless of DBS being enabled or disabled and during both rest and waiting periods of a cognitive task. We did not find any association between anxiety and theta or alpha power recorded from the electrocorticography over the premotor frontal cortex, suggesting that this relationship is region specific. Lastly, theta did not track variations in anxiety ratings across days.

To our knowledge, this is the first study to explore neural physiomarkers for non-motor symptoms using commercially available sensing-enabled DBS devices that allow chronic patient recording at home. Our approach of parallel laboratory-based and home-based assessments and recordings supports both scientific reproducibility and ecological validity.

Another feature of this study is the observation of the same relationship between basal ganglia theta and anxiety in three independent cohorts, each using somewhat different methodologies with regard to clinical setting, assessment of anxiety, and LFP recording. We studied a heterogeneous DBS population comprising unilateral and bilateral DBS of both the STN and the GPi, with LME models accounting for heterogeneity across implantation targets and laterality.

Low-frequency oscillations (including alpha and theta bands) in the STN have previously been evaluated in relationship to cognition and emotion in PD patients (for a review, see Ricciardi *et al*.^29^). Current literature implicates low frequencies in cognitive processes such as conflictual judgment,^49^ decision-making,^50^ reward-related processing^51^ and perceptual discrimination.^52^ Regarding neuropsychiatric symptoms, low frequencies have been related to trait impulsivity,^32^ ICB^30^ and depression.^53–55^

We extend this knowledge to anxiety, showing a consistent association between theta and anxiety in each of our three samples. It is, however, not possible to disentangle whether the relationship with theta is strictly specific for anxiety or whether it is driven by other highly correlated and co-occurring neuropsychiatric symptoms, especially depression.^56^ In our cohort of patients studied subacutely after surgery, theta was also positively related to depression, but importantly, this was not replicated in our two at-home cohorts, although these at-home cohorts had lower proportions of patients meeting clinical criteria for depression. Overall, our data suggest that subcortical theta might specifically index anxiety and provide some evidence that anxiety and depression might be separable neurophysiologically. Nevertheless, this remains a challenge for further research because neuropsychiatric symptoms in PD are highly co-morbid.^56^ A PD patient with anxiety is highly likely also to present with clinical symptoms of depression, ICB or apathy. Moreover, the comorbidity of anxiety and depression is highly influenced by strong overlap in diagnostic criteria and assessment scales, which might be responsible for artefactually increasing comorbidity rates,^57^ and we cannot exclude the possibility that this influences our results and previous reports. From a clinical perspective, although it has been well demonstrated that anxiety can occur independently from depression in PD,^56^ this conundrum might be less concerning for current therapeutic strategies, because treatments for the different neuropsychiatric symptoms are often similar. For example, antidepressants and psychotherapy are validated treatments for anxiety, depression and apathy in PD.^17^ However, disentangling different neuropsychiatric features in PD remains an important although challenging research question for future studies and a primary aim for developing new, effective therapeutic approaches.

Given that previous research has also linked theta/alpha power to tremor^58^ and dyskinesia,^30^ we controlled for the effects of these motor symptoms with additional analyses in the cohort of patients assessed in the hospital with externalized leads (referred to as the ‘in-hospital externalized at-rest cohort’). We found no association between tremor and alpha or theta and between dyskinesia and alpha or theta, and the relationship between theta and anxiety held when controlling for these motor symptoms in the model.

Recording LFPs via sensing-enabled DBS at home in the Percept and RC+S cohorts also enabled a comparison with daily VAS anxiety severity ratings, but we found no evidence that theta tracked these day-to-day variations in anxiety. The psychobiological sub-strates of fluctuations in anxiety captured by VAS ratings might differ substantially from standardized clinical questionnaires (e.g. BAI and HARS), which measure an average level of anxiety over the previous few weeks. The former is often referred to as ‘state’ anxiety, whereas the latter is conceptualized as ‘trait’ anxiety.^13,59^ It is therefore conceivable that ‘trait’ and ‘state’ anxiety might have different physiomarker profiles. The lack of correspondence between theta and day-to-day anxiety VAS ratings, in addition to the persistence of the relationship to clinical anxiety scores even during quiet attentive periods in the reward task and during both stimulation on and off conditions, suggest that basal ganglia theta might be more closely related to ‘trait’ anxiety.

The dissociation between state and trait anxiety in terms of physiomarkers might also reflect the heterogeneous phenomenology of anxiety in PD, which encompasses persistent and episodic anxiety or a combination of both. Also, factors such as non-motor fluctuations and situation-specific anxiety (e.g. fear of falling) are not really explored with the currently available questionnaires or ecological momentary assessment tools. This measurement gap reflects a need in future studies for more disease-specific measures to disentangle ‘state’ and ‘trait’ anxiety in PD, possibly including objective measures, such as peripheral physiology (e.g. heart rate variability, skin conductance).

A limitation of this study is the relatively small sample size, which has limited our analysis. The sample sizes of the cohorts did not allow a full, data-driven exploration of potential physiomarkers. Instead, we relied on *a priori* testing of hypotheses concerning canonical theta and alpha frequency bands, which were informed by prior literature implicating these signals in cognitive and emotional processing.^29^ However, it is worth noting that despite smaller sample sizes per cohort, this was independently replicated across three different cohorts and significant within each cohort. Our LME modelling approach included fixed effects for hemisphere and basal ganglia region to control for these potential confounds, in addition to random intercepts to account for the hierarchical nature of our repeated-measures data. However, these theoretically motivated decisions reduced statistical power to detect effects with our sample sizes, which might explain some inconsistencies across the three cohorts with regard to presence/absence of a relationship in the different stimulation and medication conditions. For example, with MedON-StimOff, there is a significant relationship between basal ganglia theta and anxiety in two of three cohorts, with a trend (β = 0.061) in the same direction in the remaining cohort. Likewise, restricted statistical power owing to sample sizes creates challenges for reliably disentangling the different contributions of hemisphere (left versus right) and target (STN versus GPi). Also, we showed an association between theta and anxiety; however, we cannot ascertain whether this reflects a primarily pathological or a secondary/compensatory process. For example, top-down communication in theta frequencies from medial prefrontal cortex to STN reflects cognitive control adjustments during uncertainty and punishment,^60–62^ and this medial frontal theta signal is stronger in individuals with higher trait anxiety.^63,64^ These data support the proposal that chronically increased anxiety might be related to excessive theta signalling of uncertainty and punishment,^65^ but the observational nature of our study precludes any inferences about brain-symptom causality and constrains our ability to assess the role of theta in the abovementioned dynamics of state anxiety.

Despite these limitations, the present study strongly suggests that basal ganglia theta could serve as an objective physiomarker of the general anxiety level in PD patients treated with DBS. Our work could potentially also provide insights into the mechanism of similar neuropsychiatric symptoms in people who do not have PD. Indeed, alpha and theta frequencies power have been linked to major depression,^66^ drug addiction,^67^ obsessive-compulsive disorder^68^ and Tourette’s syndrome,^69^ thus suggesting possible transdiagnostic networks.

More studies should confirm and extend our findings to clarify the causality of the relationship between anxiety and theta in PD. More research is also required to assess whether, within a patient, theta changes along with long-term (weeks–months) changes in general anxiety level (‘trait’ anxiety).

Similar to beta-based adaptive DBS for motor symptoms,^23,25^ basal ganglia theta could theoretically drive closed-loop paradigms aimed at providing targeted neuromodulation for anxiety. However, our present findings do not yet support the implementation of such responsive neuromodulation strategies. Unlike beta-based motor adaptive DBS, which operates on shorter time scales, potential closed-loop systems for anxiety might require adjustments for longer time scales owing to the nature of trait versus state biomarkers. Indeed, the response to DBS for depression and obsessive-compulsive disorder can require months or even years.^70–72^ To develop effective closed-loop paradigms that address short-term fluctuations in anxiety, we will need a physiomarker specifically associated with ‘state’ anxiety. Finally, the field would greatly benefit from the discovery of other objective markers of anxiety, residing in the subcortical/cortical LFP signal, autonomic system (e.g. heart rate variability,^73^ temperature or respiration changes) or elsewhere.

## Conclusion

In conclusion, we demonstrate that basal ganglia theta is related to anxiety in PD patients treated with DBS. This objective marker of anxiety could have diagnostic and therapeutic implications in clinical care.

## Supplementary Material

Supplementary Material

## Figures and Tables

**Table 1:** Patient characteristics in the three cohorts

Characteristic	In-hospital externalized at-rest	At-home chronic at-rest	At-home chronic task
Patients (*n*)	12	13	8
Hemispheres (*n*)	24	20	15^a^
Target (*n* hemispheres), STN | GPi	24 | 0	14 | 6	8 | 7
Sex (*n* patients), female | male	8 | 4	2 | 11	1 | 7
Age (years), mean (SD)	62.5 (3.9)	64.9 (9.8)	61.9 (9.6)
Use of anxiolytics and antidepressants	1/12; 0/12	0/13; 8/13	1/8; 6/8
Disease duration (years), mean (SD)	11.2 (5.6)	11.3 (4.2)	11.4 (5.7)
DBS duration (years), mean (SD)	N/A	1.5 (1.8)	2.5 (1.1)
Anxiety		
BAI, mean (SD)	N/A	9.9 (9.3)	10.5 (3.5)
HARS, mean (SD)	9.2 (7.6)	N/A	N/A
Depression		
BDI, mean (SD)	N/A	6.2 (3.7)	9.8 (8.0)
HDRS, mean (SD)	8.4 (5.4)	N/A	N/A

BAI = Beck Anxiety Inventory; BDI = Beck Depression Inventory; GPi = globus pallidus pars interna; HARS = Hamilton Anxiety Rating Scale; HDRS = Hamilton Depression Rating Scale; N/A = not applicable; SD = standard deviation; STN = subthalamic nucleus.

a Each with a quadripolar electrocorticography paddle over the sensorimotor cortex.

## Data Availability

Individual patient data fall under the health data category of the General Data Privacy Regulation and require the lawful definition of data sharing agreements from all data controllers. Data sharing agreements can be set in place upon reasonable request to the senior authors (S.L. and L.R.).
