# Supplementary material for: Basal ganglia theta power indexes trait anxiety in people with Parkinson’s disease
Source: Brain. Author manuscript; Available in PMC 2025 Mar 20. (PMC7617510; doi:10.1093/brain/awae313)
Supplement: Supplementary Material [file EMS203629-supplement-Supplementary_Material.pdf]

Supplementary data

Supplementary Methods

At-home chronic task cohort

In summary, the task (which is described in detail in[1]) is a modified version of the two-step reward learning task designed to separate habitual and goal-directed decision making strategies. On each trial, participants are randomly assigned to one of two initial states. They then choose between two options (rockets), which deterministically lead them to one of two possible second-stage states (planets). Participants receive binary reward feedback (one or zero pieces of space treasure) based on contingencies that change from trial to trial, such that reversals in reward values of each planet necessitate ongoing learning.

DBS electrodes location

The position of the leads and contacts was reconstructed using the Lead-DBS toolbox (version 3.0) (https://www.lead-dbs.org/) and imported into MATLAB R2019b (Supplementary Figure 6). CT scans containing lead location information were co-registered to pre-operative T1-weighted MRI using Advanced Normalisation Tools, then transformed into MNI space. If the automatic delineation of the STN/GPi was visibly off, the non-linear warps were manually refined using the WarpDrive tool[2]. For electrode reconstruction, leads were first automatically pre-reconstructed using the PaCER toolbox[3], then were manually refined upon visual inspection.

In the 'In-hospital externalized at-rest cohort' the average bipolar-mode LFP from electrode contact pairs within 2 mm of the motor STN was used to analyze neural oscillatory activity in the STN. All patients except one had both leads in target, and the recording contacts within (or < 2 mm from) the motor STN, therefore LFP recorded from all contacts for both leads were included in the analysis. In one patient, one lead had only two of four electrodes within 2 mm of the motor STN, hence only LFP recorded from these 2 contacts were chosen for further analysis in this patient (Supplementary Figure 1, panel A).

In the 'at-home chronic task' cohort, analyses included all bipolar LFP recordings using the two contacts immediately surrounding the stimulation site to minimize electrical artifacts. All STN

recording contacts were within 2 mm of the sensorimotor STN except the dorsal-most contact of one bipolar pair (2.30 mm away). For the GPi patients, all ventral recording contacts and one dorsal recording contact were within 2 mm of the GPi, and the remaining three dorsal recording contacts were 3.53 +/- 0.38 mm (mean +/- SD; min 3.13, max 4.12) away from the GPi, primarily in the external segment of the globus pallidus (Supplementary Figure 1, panel B). Imaging data were not readily available for lead localization in the 'at-home chronic at-rest' cohort.

Citation diversity statement

Recent work in several fields of science has identified a bias in citation practices such that papers from women and other minority scholars are under-cited relative to the number of such papers in the field[4–7]. Here we sought to proactively consider choosing references that reflect the diversity of the field in thought, form of contribution, gender, race, ethnicity, and other factors. First, we obtained the predicted gender of the first and last author of each reference by using databases that store the probability of a first name being carried by a woman[4]. By this measure and excluding self-citations to the first and last authors of our current paper), our references contain 11.71% woman(first)/woman(last), 22.22% man/woman, 28.56% woman/man, and 37.51% man/man. This method is limited in that a) names, pronouns, and social media profiles used to construct the databases may not, in every case, be indicative of gender identity and b) it cannot account for intersex, non-binary, or transgender people. Second, we obtained a predicted racial/ethnic category of the first and last author of each reference by databases that store the probability of a first and last name being carried by an author of color[8,9]. By this measure (and excluding self-citations), our references contain 12.62% author of color (first)/author of color(last), 18.87% white author/author of color, 14.96% author of color/white author, and 53.55% white author/white author. This method is limited in that a) names and Florida Voter Data to make the predictions may not be indicative of racial/ethnic identity, and b) it cannot account for Indigenous and mixed-race authors, or those who may face differential biases due to the ambiguous racialization or ethnicization of their names. We look forward to future work that could help us to better understand how to support equitable practices in science.

Supplementary Results

Patients clinical characteristics

In the ‘in-hospital externalized at-rest’ cohort, 10/12 patients presented minimal-to-mild anxiety (HARS score < 14) and 2 had moderate anxiety (HARS score 15-23) (Matza et al, IJMPR, 2010). In the ‘at-home chronic at rest’ cohort, 6/13 had minimal anxiety (BAI score < 8), 3/13 had mild anxiety (BAI score 8-15), 3/13 had moderate anxiety (BAI score 16-25), and 1/13 had severe anxiety (BAI score > 25) [10]. In the ‘at-home chronic task’ cohort, 3/8 had minimal anxiety and 5/8 had mild anxiety as per their BAI scores.

Six out of 12 patients in the ‘in-hospital externalized at-rest’ cohort scored above the cut-off for clinical depression (HDRS score > 8, [11]). In the 'at-home chronic at rest' cohort, none of the patients scored above the cut-off for clinical depression (BDI score >13, [12]) while in the ‘at-home chronic task’ cohort 1/8 patients (12.5%) scored above the cut-off. Chi-square analysis showed that the proportion of subjects who reported being depressed was not significantly different between the ‘in-hospital externalized at-rest cohort’ and the 'at-home chronic at-rest cohort' ($X^2 = 3.5$, $p = 0.06$). Whereas there was a significant difference in the proportion of subjects with depression between the ‘in-hospital externalized at-rest’ cohort and the ‘at-home chronic at-rest’ cohort ($X^2 = 4.2$, $p = 0.04$).

Relationship between depression and low frequencies activity

In the ‘in-hospital externalized at-rest’ cohort, depression (as per HDRS score) was positively related to STN alpha and theta band power in the MedOFF StimOFF condition (p = 0.025 and β = 0.067 for alpha, p = 0.018 and β = 0.067 for theta), but not with MedON StimOFF (p = 0.716 and β = 0.034 for alpha, p = 0.439 and β = 0.019 for theta) (Supplementary Fig. 6, panel A). In the ‘at-home chronic at-rest’ cohort, depression (as per BDI) was positively related to basal ganglia alpha band power in MedON StimOFF (p = 0.031, β = 0.005), but not with MedON StimON (p = 0.620, β < 0.001) (Supplementary Fig. 6, panel B). In this cohort, depression was not related to basal ganglia theta band power (all p > 0.072, all β < 0.005) (Supplementary Fig. 6, panel B). In the ‘at-home chronic task’ cohort, depression (as per BDI) was not related to basal ganglia alpha nor theta band power in any of the conditions (all p > 0.05) (Supplementary Fig. 6, panel C).

Supplementary Figures

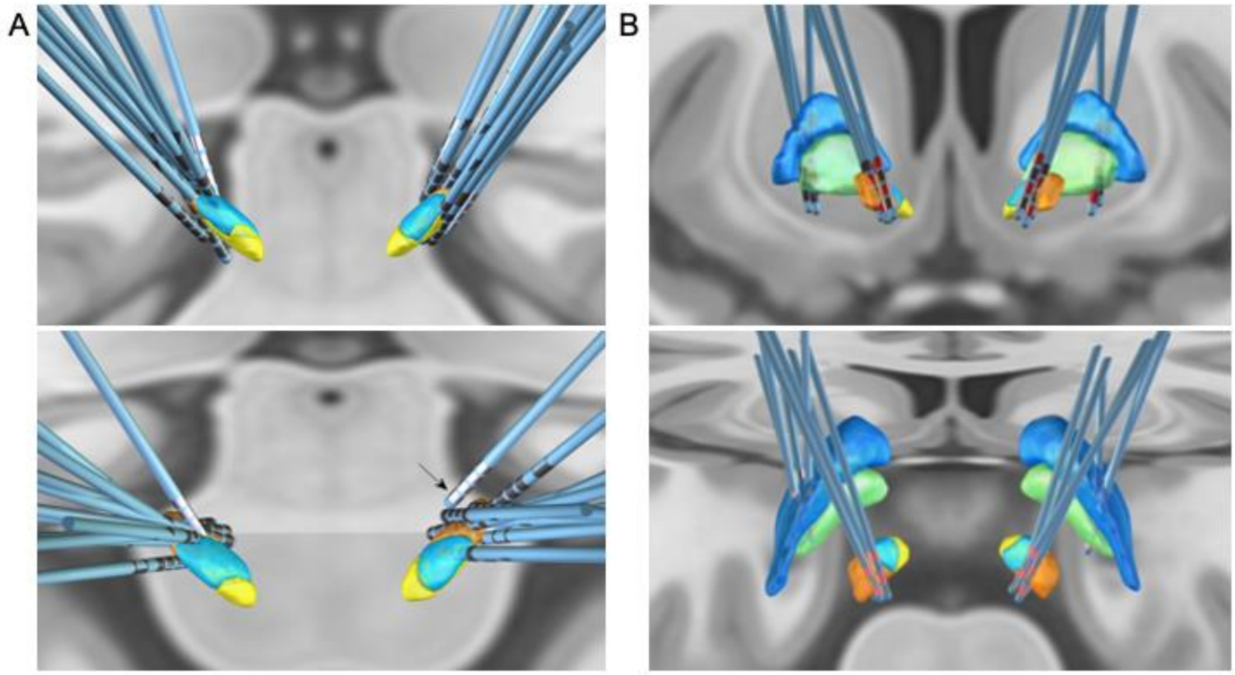

Supplementary figure 1. Localization of electrode leads for two cohorts. Coronal (top) and axial (bottom) views of electrode lead locations in MNI space. Panel A includes patients of the 'in-hospital externalized at-rest' cohort (n=12). All contacts were within 2 mm of the sensorimotor STN, except for the two dorsal-most contacts on one lead (white contacts). Panel B shows results of the 'at-home chronic task' cohort (n=4 STN, n=4 GPi). LFPs were recorded from bipolar pairs of contacts highlighted in red, which were chosen in "sandwich" configuration on either side of the stimulation contacts to minimize artifacts. All STN recording contacts were within 2 mm of the sensorimotor STN except the dorsal contact of one bipolar pair (2.30 mm away). For the GPi patients, all ventral recording contacts and one dorsal recording contact were within 2 mm of the GPi, and the remaining three dorsal recording contacts were 3.53 +/- 0.38 mm (mean +/- SD; min 3.13, max 4.12) away from the GPi, primarily in the external segment of the globus pallidus.

Color legend (subregions from the Distal Minimal Atlas [13]): Panels A and B: orange, STN sensorimotor, light blue, STN associative, yellow, STN limbic. Panel B: green, globus pallidus internal segment, darker blue, globus pallidus external segment.

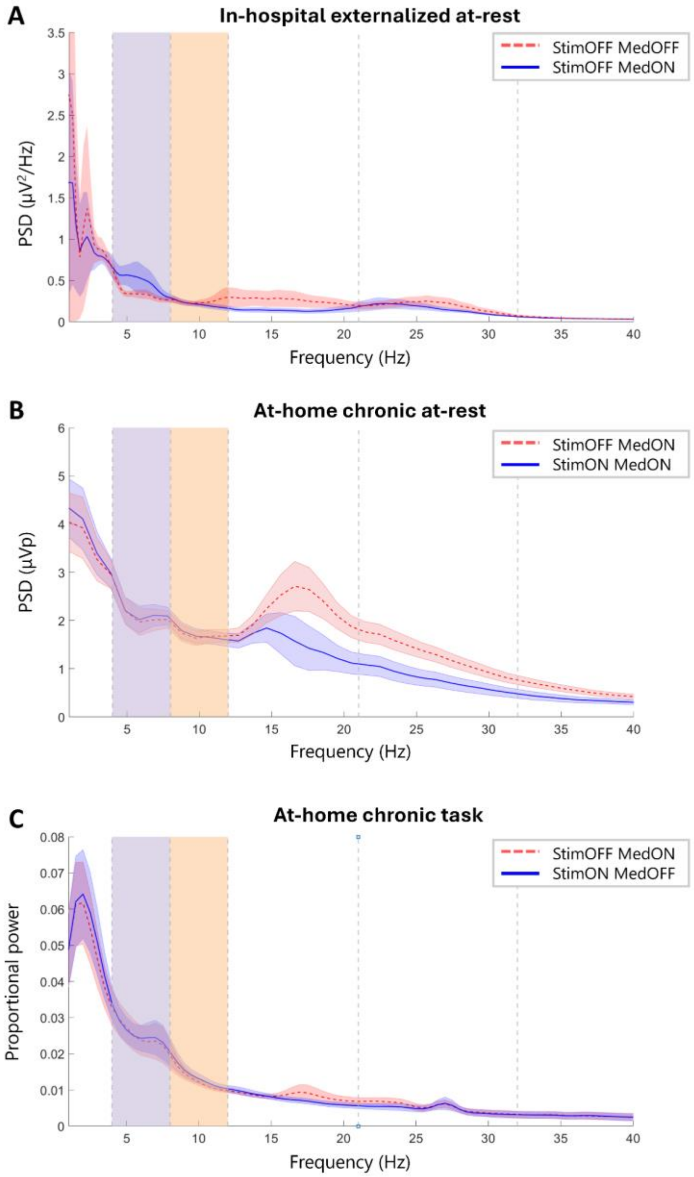

Supplementary figure 2. Effect of medication and stimulation on the power frequency spectrum across the three cohorts. Each panel represents the power spectrum density of a cohort in the different conditions of medication/stimulation. Theta (4-8 Hz) and alpha (8-12 Hz) bands are highlighted in violet and orange, respectively. Vertical dashed lines highlight low-beta (12-21 Hz), high-beta (21-32 Hz), and gamma (32-40 Hz) bands. In the 'in-hospital externalized at-rest' cohort (panel A), no statistically significant effect of medication is found in alpha, high-beta, or gamma bands. The effect of medication trends towards statistical significance in theta ($p = 0.05$, lower values in the MedOFF condition) and low-beta bands ($p = 0.07$, higher values in the MedOFF condition). In the 'at-home chronic at-rest' cohort (panel B) lower power values are

found in the StimON MedOn condition compared to the StimOFF MedON condition for low-beta, high-beta, and gamma frequency ranges (all $p < 0.0001$). No stimulation effect is present in theta and alpha bands. In the ‘at-home chronic task’ cohort (Panel C), no statistically significant effect of the stimulation is found in any frequency band.

Color legend: Panel A: red, StimOFF MedOFF, blue, StimOFF MedON. Panel B: red, StimOFF MedON, blue, StimON MedON. Panel C: red, StimOFF MedON, blue, StimON MedOFF. Abbreviations: PSD; power spectrum density.

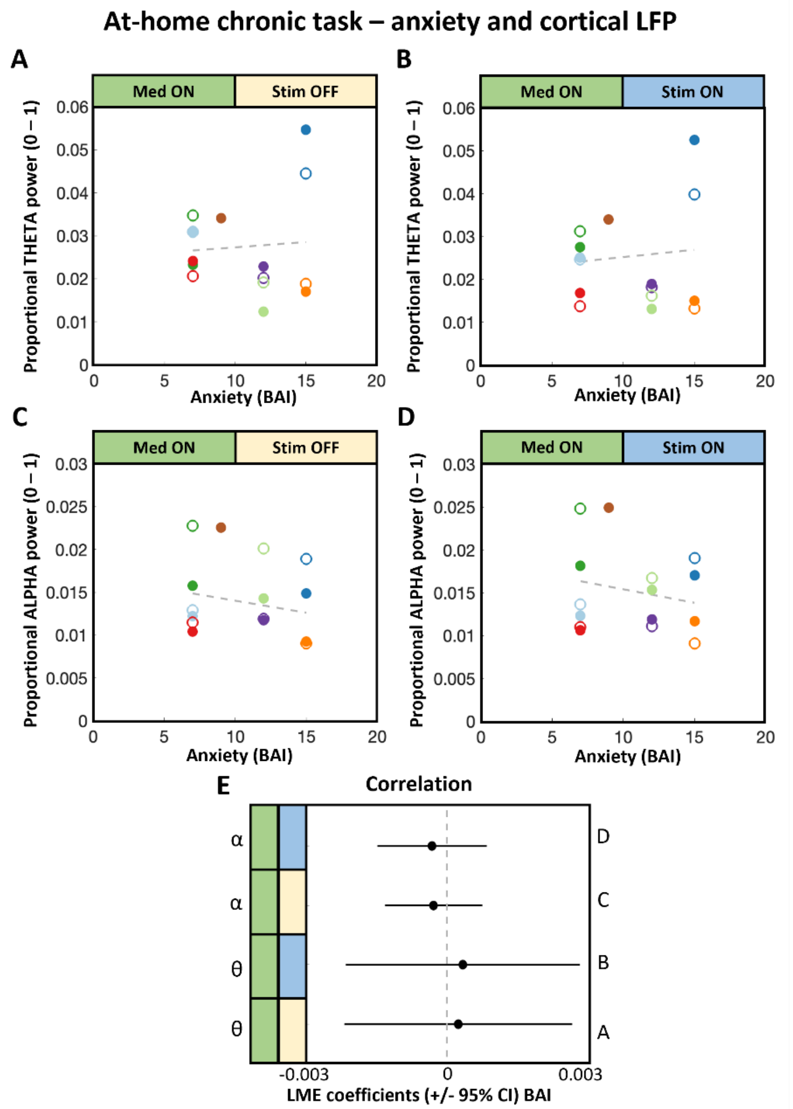

Supplementary figure 3. Anxiety is not related with premotor cortical theta or alpha. Relation between anxiety and premotor cortical LFP during a reward task in the 'at-home chronic task' cohort. Theta and alpha power were extracted from premotor electrocorticography recordings during the inter-trial interval in a reward task, and correlation was assessed with baseline anxiety (BAI). Scatter plot colors indicate participant (n = 8 patients), with circles indicating STN and squares indicating GPi implants. Empty/filled markers indicate right/left hemisphere recordings (n = 15 hemispheres). In none of the medication/stimulation conditions (*p* values for Panels A to D: 0.83, 0.77, 0.56, 0.56), anxiety is related to cortical theta or alpha band power. Panel E illustrates the coefficients of BAI in LME models. Abbreviations: BAI, Beck anxiety inventory; CI, confidence interval; LFP, local field potential; LME, linear mixed-effects model.

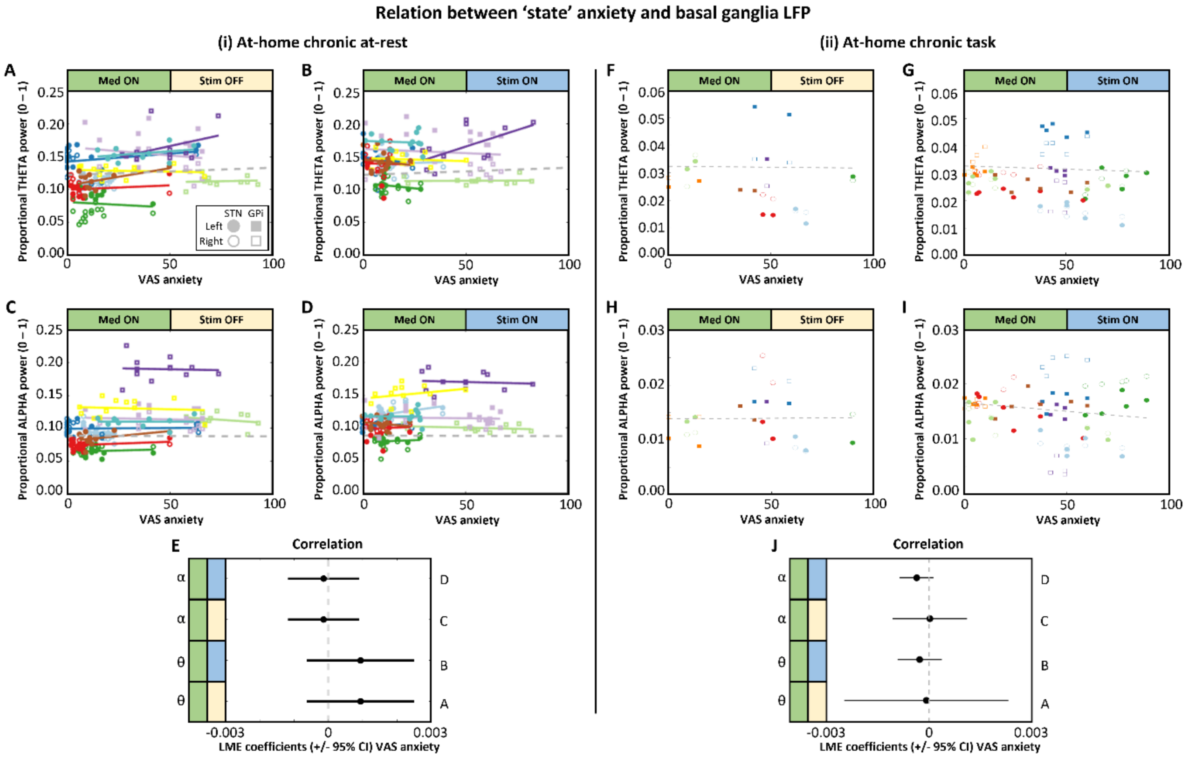

Supplementary figure 4. Basal ganglia theta and alpha do not track within-subject 'state' anxiety variation. Relation between state anxiety variation (as per VAS anxiety rating) and basal ganglia LFP in the 'at-home chronic at-rest' (panels A to E - n = 10 participants with at least five VAS ratings) and 'at-home chronic task' (panels F to J - n = 8 participants) cohorts. Scatter plot (panels A to D, and F to I) colors indicate participant, with circles indicating STN and squares indicating GPi implants, and empty/filled markers indicating right/left hemisphere recordings. Using an LME model, VAS anxiety was not related to basal ganglia theta or alpha in neither cohort (all $p > 0.143$, all $\beta < 0.0001$). Panels E and J illustrate the coefficients of VAS anxiety in LME models. Solid colored lines in panels A to D represent linear regression trendlines per participant - for visual representation purpose. Abbreviations: CI, confidence interval; LFP, local field potential; VAS, visual analogue scale.

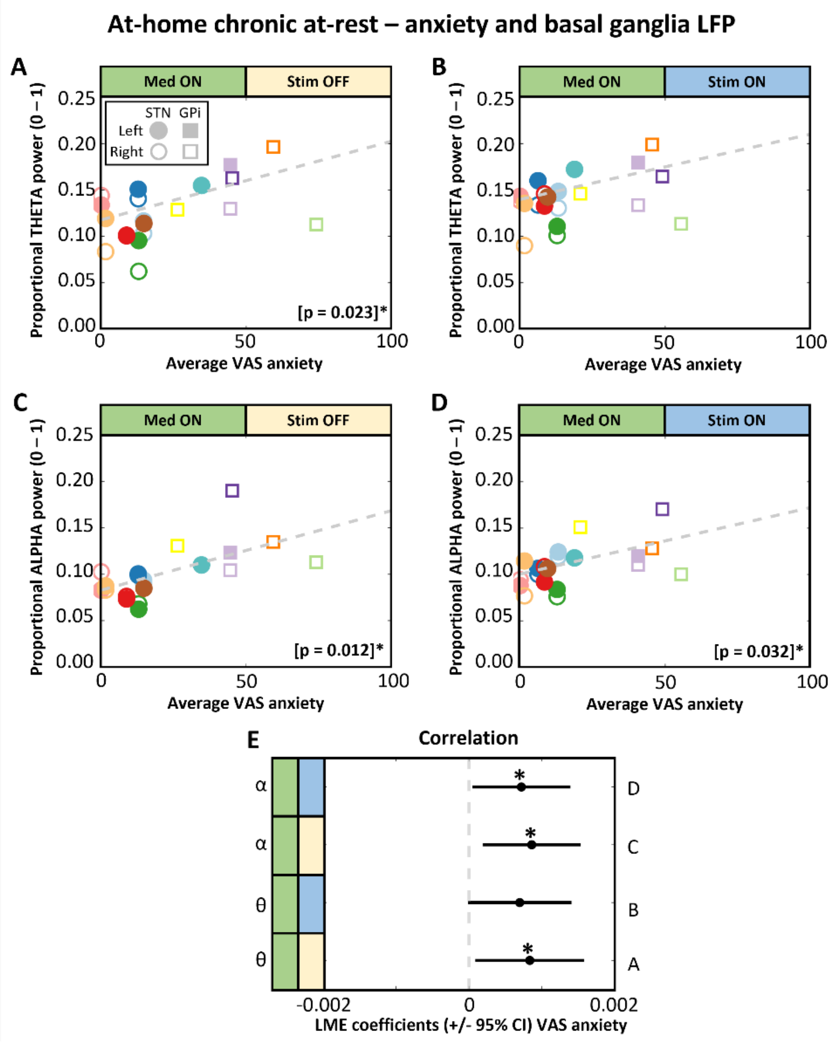

Supplementary figure 5. Average 'state' anxiety is related to basal ganglia theta and alpha in the at-home unsupervised environment. Relation between average state anxiety and subcortical LFP in the 'at-home chronic at-rest' cohort. Scatter plot colors indicate participant (n = 13 patients), with circles indicating STN and squares indicating GPi implants. Empty/filled markers indicate right/left hemisphere recordings (n = 20 hemispheres). Per hemisphere, theta and alpha band power were extracted from at-home at-rest BrainSense Event LFP recordings. Correlation was assessed with the, per patient, average momentaneous anxiety level measured over 14 days via a visual analogue scale (VAS). With Medication ON Stimulation OFF, anxiety is positively related with subcortical theta (panel A) and alpha (panel C) band power. With Medication ON Stimulation ON, anxiety is positively related with subcortical alpha (panel D) band power. Panel E illustrates the coefficients of BAI in LME models. *, $p < 0.05$. Abbreviations: CI,

confidence interval; GPi, globus pallidus pars interna; LFP, local field potential; LME, linear mixed-effects model; STN, subthalamic nucleus; VAS, visual analogue scale.

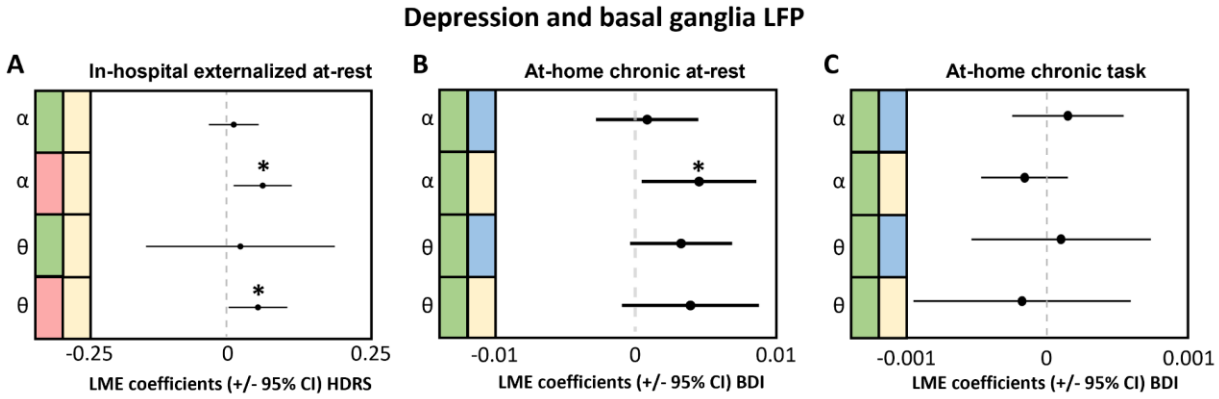

Supplementary figure 6. Relation between depression and basal ganglia LFP across three cohorts. Each panel illustrates the coefficients of depression (as per HDRS or BDI) in LME models assessing the relation between depression and basal ganglia LFP within each cohort. With medication and stimulation OFF, basal ganglia theta and alpha band power are positively related with depression (as per HDRS) in the 'in-hospital externalized at-rest' cohort (panel A). With medication ON and stimulation OFF, basal ganglia alpha band power is positively related with depression (as per BDI) in the 'at-home chronic at-rest' cohort (panel B). In the 'at-home chronic task' cohort, depression is not related to basal ganglia theta or alpha power with medication ON and stimulation ON or OFF (panel C). *, $p < 0.05$. Color legend: green, Medication ON; red, Medication OFF; blue, Stimulation ON; yellow, Stimulation OFF. Abbreviations: BDI, Beck depression inventory; CI, confidence interval; HDRS, Hamilton depression rating scale; LFP, local field potential; LME, linear mixed-effects model.

Supplementary References

1. Kool W, Cushman FA, Gershman SJ. When Does Model-Based Control Pay Off? *PLoS Comput Biol*. 2016;12(8):e1005090.

2. Oxenford S, Ríos AS, Hollunder B, et al. WarpDrive: Improving spatial normalization using manual refinements. *Med Image Anal*. 2024;91:103041.

3. Husch A, Petersen M V, Gemmar P, Goncalves J, Hertel F. PaCER - A fully automated method for electrode trajectory and contact reconstruction in deep brain stimulation. *Neuroimage Clin*. 2018;17:80-89.

4. Dworkin JD, Linn KA, Teich EG, Zurn P, Shinohara RT, Bassett DS. The extent and drivers of gender imbalance in neuroscience reference lists. *Nat Neurosci*. 2020;23(8):918-926.

5. Chatterjee P, Werner RM. Gender Disparity in Citations in High-Impact Journal Articles. *JAMA Netw Open*. 2021;4(7):e2114509-e2114509.

6. Fulvio JM, Akinnola I, Postle BR. Gender (Im)balance in Citation Practices in Cognitive Neuroscience. *J Cogn Neurosci*. 2021;33(1):3-7.

7. Bertolero MA, Dworkin JD, David SU, et al. Racial and ethnic imbalance in neuroscience reference lists and intersections with gender. *bioRxiv*. Published online October 12, 2020:2020.10.12.336230. doi:10.1101/2020.10.12.336230

8. Chintalapati R, Laohaprapanon S, Sood G. Predicting Race and Ethnicity From the Sequence of Characters in a Name. Published online May 5, 2018. Accessed August 14, 2024. http://arxiv.org/abs/1805.02109

9. Name-ethnicity classification from open sources. doi:10.1145/1557019.1557032

10. Schlaepfer TE, Nemeroff CB. *Neurobiology of Psychiatric Disorders*. Elsevier; 2012.

11. Zimmerman M, Martinez JH, Young D, Chelminski I, Dalrymple K. Severity classification on the Hamilton Depression Rating Scale. *J Affect Disord*. 2013;150(2):384-388.

12. T. BA. *Beck Depression Inventory (BDI-II), Second Edition. San Antonio, TX: The Psychological Corporation, 1996*.

13. Ewert S, Plettig P, Li N, et al. Toward defining deep brain stimulation targets in MNI space: A subcortical atlas based on multimodal MRI, histology and structural connectivity. *Neuroimage*. 2018;170:271-282.
